# The Shape of Things To Come: The Formation of Modulated Nematic Mesophases at Various Length Scales

**DOI:** 10.1002/chem.201701167

**Published:** 2017-06-05

**Authors:** Richard J. Mandle

**Affiliations:** ^1^ Department of Chemistry University of York, Heslington York YO10 5DD UK

**Keywords:** hydrogen bonds, liquid crystals, oligomers

## Abstract

The twist–bend nematic (N_TB_) phase is a recently discovered liquid‐crystalline phase that exhibits macroscopic chirality even when formed from achiral materials, and as such presents a unique testbed for studies concerning the spontaneous breaking of mirror symmetry in soft matter. It is primarily exhibited by materials for which the molecular structure is composed of two rigid aromatic units (such as biphenyl connected by a flexible spacer). The local structure of the N_TB_ phase is nematic‐like—with molecules having an average orientational order but no positional order—with a nanoscale helix in which the pitch (i.e., the repeat distance of the helix) is of the order of several nanometres. A helix is chiral, and so the bulk N_TB_ phase—in the absence of a biasing chiral environment—spontaneously separates into macroscopic domains of opposite handedness. After discussing the structure of this mesophase and its elucidation, this concept article presents the molecular factors that determine its incidence. The apparent dependency primarily on molecular shape and bend angle rather than particular functional group combinations manifests in this mesophase being exhibited on length scales far beyond those of simple liquid‐crystalline dimers, not only in oligomers and polymers, but also in aqueous suspensions of micron sized helical particles.

## Introduction

**Figure 1:**
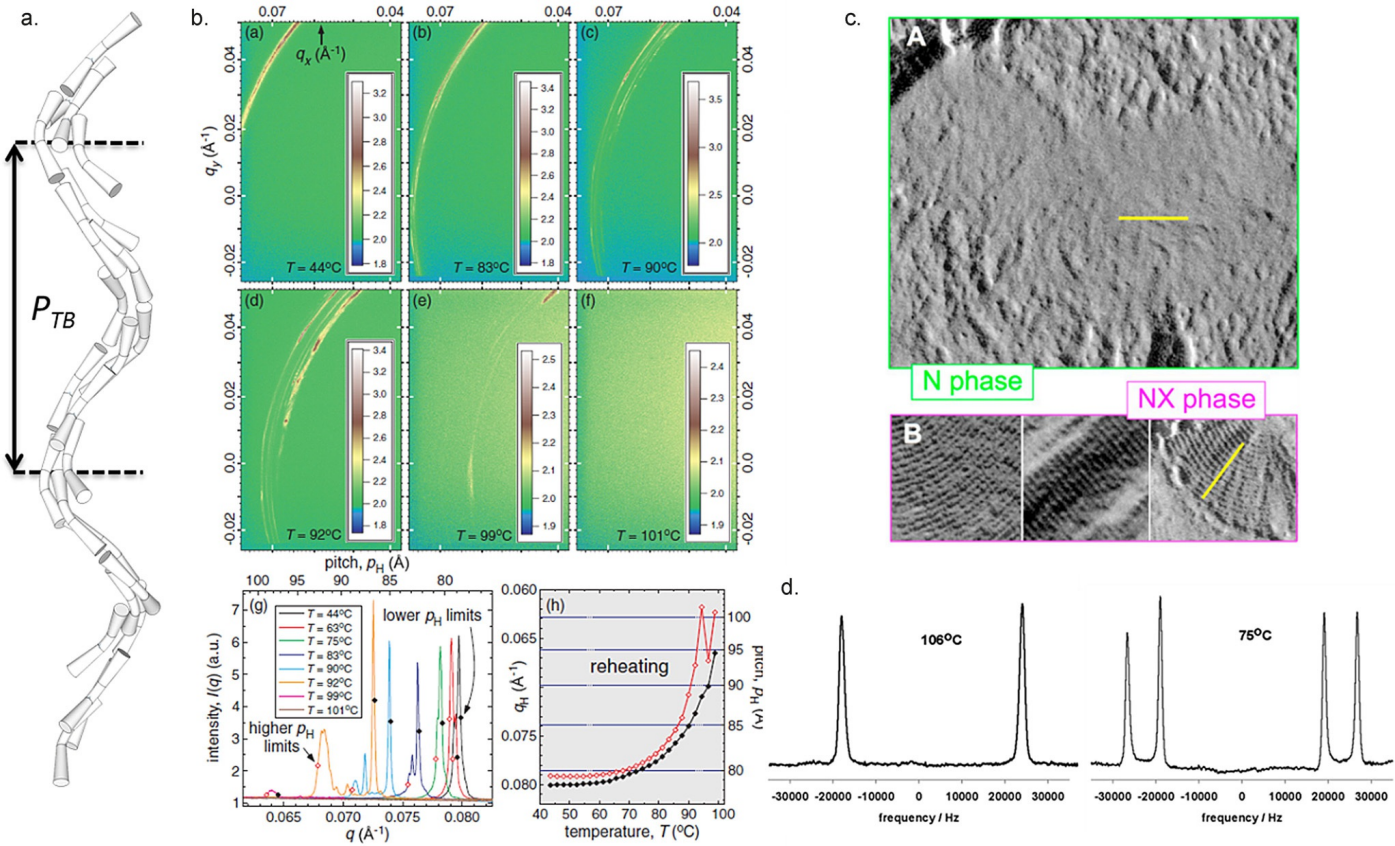
The twist–bend nematic phase: a) Cartoon depiction of a bent, U‐shaped dimers with a bend angle of ≈110° forming a N_TB_ phase whose pitch (*P*_TB_) is about 3.5 times the dimer length. b) Resonant small‐angle X‐ray scattering at the carbon K‐edge (*E*=283.5 eV) of the dimer CB9CB at various temperatures in the N_TB_ and classical nematic mesophases on a silicon nitride surface, reprinted from reference 9, copyright 2016 by the American Physical Society. c) Comparison of the freeze–fracture TEM image of CB7CB in the nematic phase quenched at 105 °C (A) and the N_TB_ phase quenched at 95 °C (B, labelled as N_X_)—in both mesophases the scale bar corresponds to 100 nm (reproduced from reference 7, used with permission of the National Academy of Sciences of the United States of America, copyright 2013).^7^ d) ^1^H NMR spectra of [D_2_]8CB dissolved in CB7CB and recorded in the nematic (106 °C, 46.0 MHz) and twist–bend nematic (75 °C, 61.4 MHz), adapted with permission from reference 15, copyright American Chemical Society 2012.^15^

The twist–bend nematic phase (N_TB_, also referred to as twist–bend phase or TB) presents perhaps one of the most well‐understood examples of spontaneous breaking of mirror symmetry in soft matter. In the N_TB_ phase there exists a local helical structure of very short pitch of about 10 nm.^1^ In the N_TB_ phase the molecules are tilted with respect to the helix axis, but lack positional ordering and thus the mesophase is “nematic”.^2^ Similarly, other modulated nematic‐like mesophases have been predicted to occur (splay–bend nematic (N_SB_),^3^, ^4^ and screw nematic (N_S_*)^5^, ^6^ to give two examples.) The pitch length of the N_TB_ helix (*P*_TB_) has been directly measured by freeze–fracture transmission electron microscopy,^1^, ^7^, ^8^ by resonant carbon K‐edge small‐angle X‐ray scattering^9^ and by resonant selenium small‐angle X‐ray scattering.^10^ All three methods give a qualitative measurement of the pitch length, which is of the order of several nanometers. Deuterium NMR spectroscopy conducted in situ on samples doped with a suitable spin probe has also been interpreted as supporting the presence of a local helix with a nanoscale pitch (i.e., several molecular lengths),^11^ although other models have been used to interpret these results.^12^, ^13^ Examples of FFTEM, RoSAXS and ^2^H NMR data are given in Figure 1. These methods have proved invaluable in the study of the N_TB_ phase, providing the strongest evidence yet for the presently accepted model of this phase, and it is to be expected that they will find great utility in the study of other modulated nematic and smectic mesophases as they arise. For example, in addition to the N_TB_ and N_SB_ mesophases already mentioned, an extension of Landau–de Gennes theory of nematics has been used to predict the existence of two as yet experimentally undiscovered polar nematic phases with transverse (N_TP_) and longitudinal (N_LP_) polarisation.^14^

**Figure 2:**
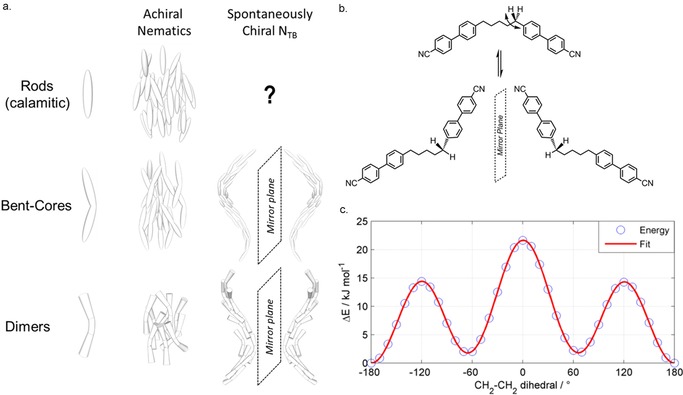
Spontaneous breaking of mirror symmetry and chiral conformational isomerism: a) Cartoon depictions of the local molecular organisation present in the achiral uniaxial nematic phase and the spontaneously chiral twist–bend nematic phase exhibited by rod‐like (calamitic), bent core and dimeric liquid crystals. b) Demonstration of rotation about the first ‐CH_2_‐CH_2_‐ dihedral in a cyanobiphenyl dimer with a pentamethylene spacer (CB5CB, transitions: Cr 150 N_TB_ 92 N 97 Iso) leading to −*gauche* (Figure 2 c −60 °) and +*gauche* (Figure 2 c +60 °) conformers, which are non‐superimposable. c) Plots of energy (kJ mol^−1^) versus this torsional angle as obtained for an isolated molecule of CB5CB using relaxed scans (36×10° steps) at the B3LYP/6‐31G(d) level of DFT in the Gaussian 09 suite of programs.^20^ The solid red line is a fit to guide the eye.

A helix is inherently chiral and so the helical N_TB_ phase spontaneously separates into macroscale domains of opposite handedness when formed from an achiral material. Due to their flexibility liquid crystal (LC) dimers can adopt a range of conformations, some of which—for example a single *gauche* in the alkyl chain—will be chiral. In the absence of a biasing chiral environment, such conformers are expected to occur in pairs that are separated by a mirror plane (Figure 2 b) with equal probability, thus the conformationally averaged structure is achiral. The +/− *gauche* conformers of CB5CB (1,ω‐bis(4‐cyanobiphenyl‐4′‐yl)pentane) are higher in energy than the *trans* conformers by about 1.8 kJ mol^−1^ at the B3LYP/6‐31G(d) level of DFT with a rotational barrier of about 14.4 kJ mol^−1^ (Figure 2 c), which implies that there is rapid interconversion between these states within the temperature range of interest (300–450 K for the 1,ω‐bis(4‐cyanobiphenyl‐4′‐yl)alkane (CB*n*CB) materials).^16^, ^17^ In the case of a biasing chiral environment such as the presence of a chiral additive, or when the material that exhibits the phase is itself chiral, only one hand of the N_TB_ helix forms.^18^ It has been demonstrated that the local chirality of the N_TB_ phase results in a small chiral biasing of the conformer distribution of bimesogens, but that the spontaneous conformational chirality *is not* the origin of the chirality of the N_TB_ phase.^19^

**Figure 3:**
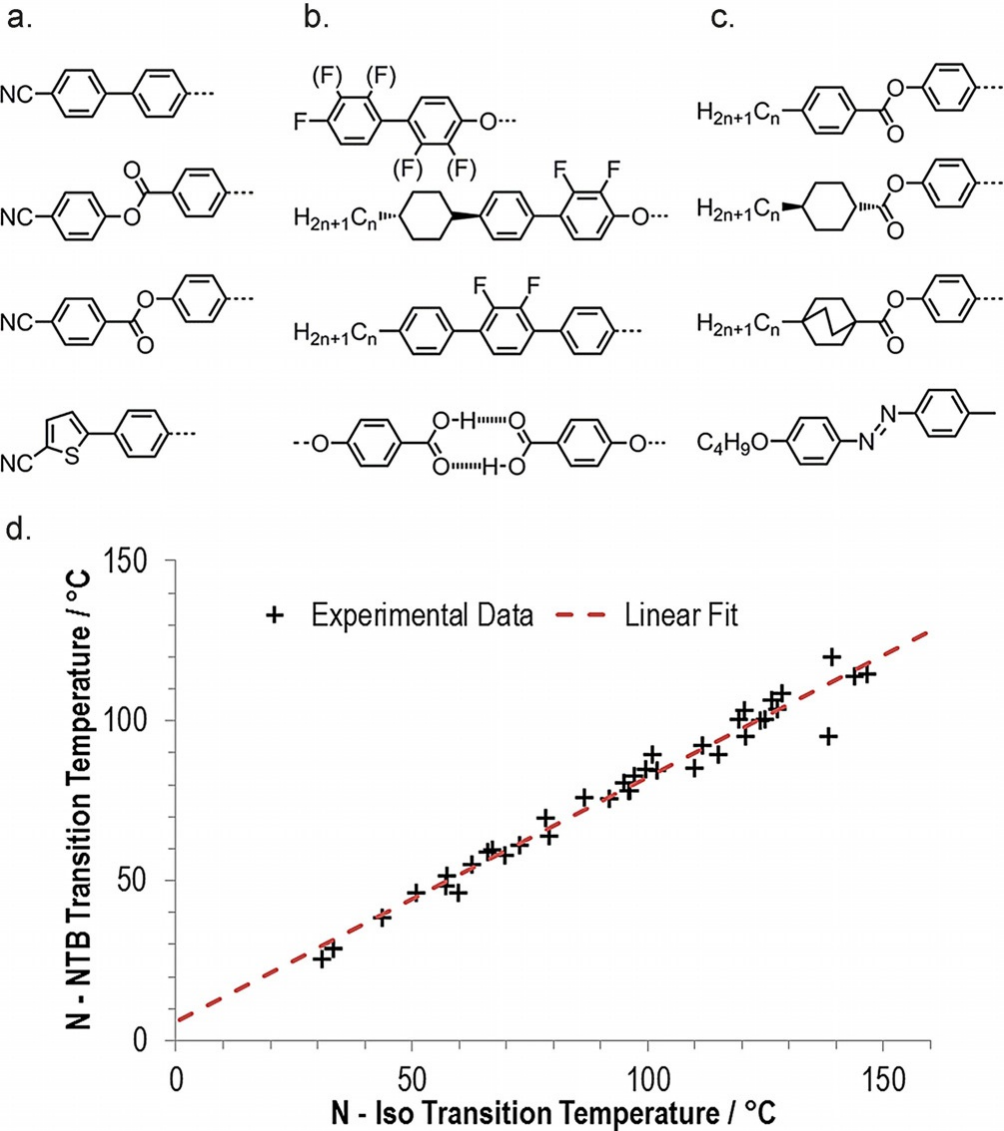
Molecular structure and the twist–bend nematic phase: a) core structures incorporating terminal nitrile units;^21^, ^28^, ^34^ b) core structures incorporating fluoro groups or hydrogen bonds;^22^, ^23^, ^24^, ^29^, ^32^ c) core structures incorporating terminal alkyl chains, saturated ring systems and azo groups.^30^, ^40^, ^41^ d) Plot of the N_TB_–N transition temperature versus the N–Iso transition temperature for dimers and bimesogens with a nonamethylene spacer, methylene linking groups and mesogenic units consisting of two aromatic or aliphatic rings. Similar plots can be constructed for oligomers, polymers, dimers containing various aspect ratios or spacer lengths and so forth, resulting in linear fits with differing slopes. The fit takes the form *T*_NTB–N_=0.766 *T*_N–Iso_+5.74 with *R* ^2^>0.97. Tabulated data was taken from reference 27.

Although initially reported only in methylene linked dimers,^21^, ^22^ the N_TB_ phase has been observed in dimers with various linking groups,^23^, ^24^, ^25^, ^26^ as well as in bent–core materials^8^ and presently there are about 140 dimeric materials known to exhibit the twist–bend nematic phase.^27^ A number of different mesogenic units have been studied within the context of the N_TB_ phase as shown in Figure 3 a–c. In addition to the well‐studied cyanobiphenyl derivatives, the N_TB_ phase has been observed for materials with mesogenic units incorporating heterocycles,^28^ laterally fluorinated rings,^22^, ^29^ cyclohexyl and bicyclohexyl rings,^30^ photoisomerisable azo‐linkers,^31^ and trimeric systems formed from hydrogen‐bonded dimers of benzoic acid derivatives.^32^ The largest sub‐grouping within this number is materials possessing methylene linking groups, a nonamethylene spacer and two mesogenic units comprised of two rigid cyclic units (40 in total).^33^ The bias towards this subdivision is a product of availability of chemical reagents rather than some advantage conferred by this particular combination of structural features. We reported previously for this subdivision, and indeed all comparable divisions that we are aware of, that a linear relationship exists between the N_TB_–N and N–Iso transition temperatures (Figure 3 d).^33^ This result complements earlier studies that found no correlation between the incidence of this phase and molecular properties, such as polarisability or electric dipole moment.^30^, ^34^ Such an outcome was first noted in a theoretical treatment by Greco et al.^35^ and later Vaupotic et al. proposed that “the internal structure of the TB nematic is driven mainly by steric interactions.”^36^ Experimental results would appear to be in agreement with these theoretical treatments; the N_TB_ phase is driven by molecular shape and steric interactions rather than any combination of functional groups and so forth.^37^ The twist–bend nematic phase can be observed for mixtures between a dimer (or bimesogen, trimesogen etc.) and a rod‐like molecule. Tuchband et al. have demonstrated that for mixtures of the dimer CB7CB with the rod‐like 5CB (4‐pentyl‐4′‐cyanobiphenyl) both the mean N_TB_ pitch length and distribution of pitch lengths (measured by FFTEM^38^ and carbon K‐edge RoSAXS^39^) increases with increasing concentration of the rod‐like component. As CB7CB is “bent” and 5CB is rod‐like the increase in pitch length as a function of 5CB concentration may indicate a relationship between the molecular bend angle and the periodicity of the N_TB_ helix.^39^

The odd parity of the central spacer found in all N_TB_ dimers confers a bent molecular shape, and theoretical treatments indicate that the thermal stability of the N_TB_ phase should display a dependency on just how bent (or not) the molecular structure of a given material is.^42^ In 2016 we reported that the angle between the two mesogenic units is one of the prime factors in determining the thermal stability of the twist–bend nematic phase.^43^ These investigations were undertaken, in part, to see if theoretical predictions of a link between the bending angle and the N_TB_ phase would be borne out in experimental work. Bend angles were taken to be the all‐*trans* conformer that we found to be dominant using solution‐based 1D ^1^H NOESY NMR spectroscopy; indeed this is also reported to be the dominant conformer in the bulk N_TB_ phase from ^2^H NMR data coupled with DFT calculations.^19^ The dominance of the all‐*trans* conformer over other conformations has been demonstrated for both methylene‐ and ether‐linked dimers using proton‐enhanced local field (PELF) NMR spectroscopy on CB7CB^19^ and dielectric spectroscopy on 1′′‐(2′,4‐difluorobiphenyl‐4′‐yloxy)‐9′′‐(4‐cyanobiphenyl‐4′‐yloxy)nonane (FFO9OCB), respectively.^44^ In reality the use of a single conformer to describe the bend angle is insufficient for flexible molecules, such as LC dimers, and so we opted to study a new set of materials computationally, obtaining a library of conformers from which we can then obtain the full distribution of bend angles as well as a weighted average molecular bend.^45^ Table 1 shows the transition temperatures of some cyanobiphenyl dimers with varying linking group and spacer composition. Some values were obtained from a survey of the literature (compounds **3**,^17^ **6**,^46^ **7**,^31^ **8** 43), whilst novel compounds were synthesised (compounds **1**, **2**, **4**, **5**).^45^

**Table 1:** Transition temperatures [°C] for compounds **1**–**7**, with the trivial names of **3** and **6** also given. Values were taken from.^17^, ^43^, ^45^, ^46^ *.

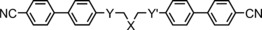										
	X	Y	Y′	Cr		N_TB_		N		Iso
**1**	‐CH_2_‐	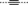	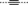	•	160.5	–	–	–	–	•
**2**	‐O‐	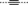	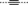	•	>225	–	–	–	–	•
**3** (CB7CB)	‐CH_2_‐	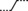	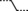	•	103.1	•	106.5	•	118.9	•
**4**	‐O‐	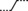	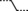	•	100.5	(•	46.0)	•	68.0	•
**5**	‐CH_2_‐	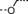	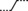	•	132.8	(•	97.0)	•	145.2	•
**6** (CB6OCB)	‐CH_2_‐	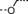	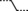	•	100.4	•	109.2	•	153.3	•
**7** (CBO5OCB)	‐CH_2_‐	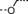	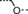	•	139	•	79^[a]^	•	187	•
**8**	‐CH_2_‐	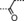	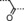	•	158.1	(•	145.1)	•	189.4	•

[a] Values from CBO5OCB were obtained by extrapolation and are taken from reference 31.

Both materials bearing two alkyne linking groups (**1** and **2**) were non mesogenic, with **2** decomposing upon heating. Paterson et al. reported 1‐(4‐cyanobiphenyl‐4′‐yloxy)‐6‐(4‐cyanobiphenyl‐4′‐yl)hexane (CB6OCB—compound **6** in this work), finding that replacing one of the two methylene groups adjacent to the 4‐cyanobiphenyl mesogenic unit in CB7CB confers a modest increase in the N_TB_–N transition temperature, *T*_NTB–N_.^46^ Positioning a single ether link in the centre of CB7CB (to give an isomer of **6**/CB6OCB) lead to the finding that *T*_NTB‐N_ drops dramatically relative to both parent materials. Increasing the rigidity of the central spacer of **6** by incorporating an alkyne unit (**5**) reduces both the clearing point and N_TB_–N transition temperatures. When two ether‐linking units are used (**7**/CBO5OCB) there is a prominent reduction in *T*_NTB–N_ relative to both compounds **3** and **6**. The use of two ketones as linking units (**8**) led to a large increase in both *T*_NTB–N_ and *T*_N–Iso_ relative to the parent compound **3**.^43^

**Figure 4:**
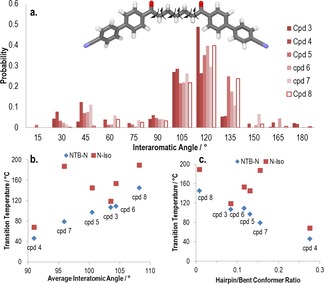
How the conformational landscape influences the twist–bend nematic mesophase: a) Top: the AM1 minimised all‐*trans* form of compound **7** with arrows showing the bonds allowed to undergo threefold rotation during the conformer search (total of 3^6^ conformers). Bottom: histogram plot of the probability of a given bend angle as determined using relaxed scans with the AM1 semi‐empirical method for compounds **3**–**8**. b) Plot of the N_TB_–N and N–Iso transition temperatures versus the Boltzmann weighted average bend angle for compounds **3**–**8**. c) Plot of the N_TB_–N and N–Iso transition temperatures versus the ratio of hairpin (defined arbitrarily as a bend angle <60 °) to bent (defined arbitrarily as a bend angle ≥60 °, <150°) conformers.^45^

Performing relaxed scans using the AM1 semi empirical method to obtain a library of conformers for compounds **3**–**8** (Figure 4 a) allows a Boltzmann weighted average bend angle to be obtained (Figure 4 b) and also the ratio between hairpin and bent conformers (bend angle <60° and bend angle ≥60°, <150° respectively) to be determined (Figure 4 c). As with previous work, this demonstrates the importance of the bend angle and the intimate relationship that exists between this and the thermal stability of the N_TB_ mesophase (i.e., the onset temperature),^43^ and reaffirms the conclusion that materials lacking an overall bent shape (i.e., even parity homologues) cannot exhibit the N_TB_ phase as it is presently understood. It is also apparent that the clearing point does not appear to display such a strong dependence on the bend angle.

**Figure 5:**
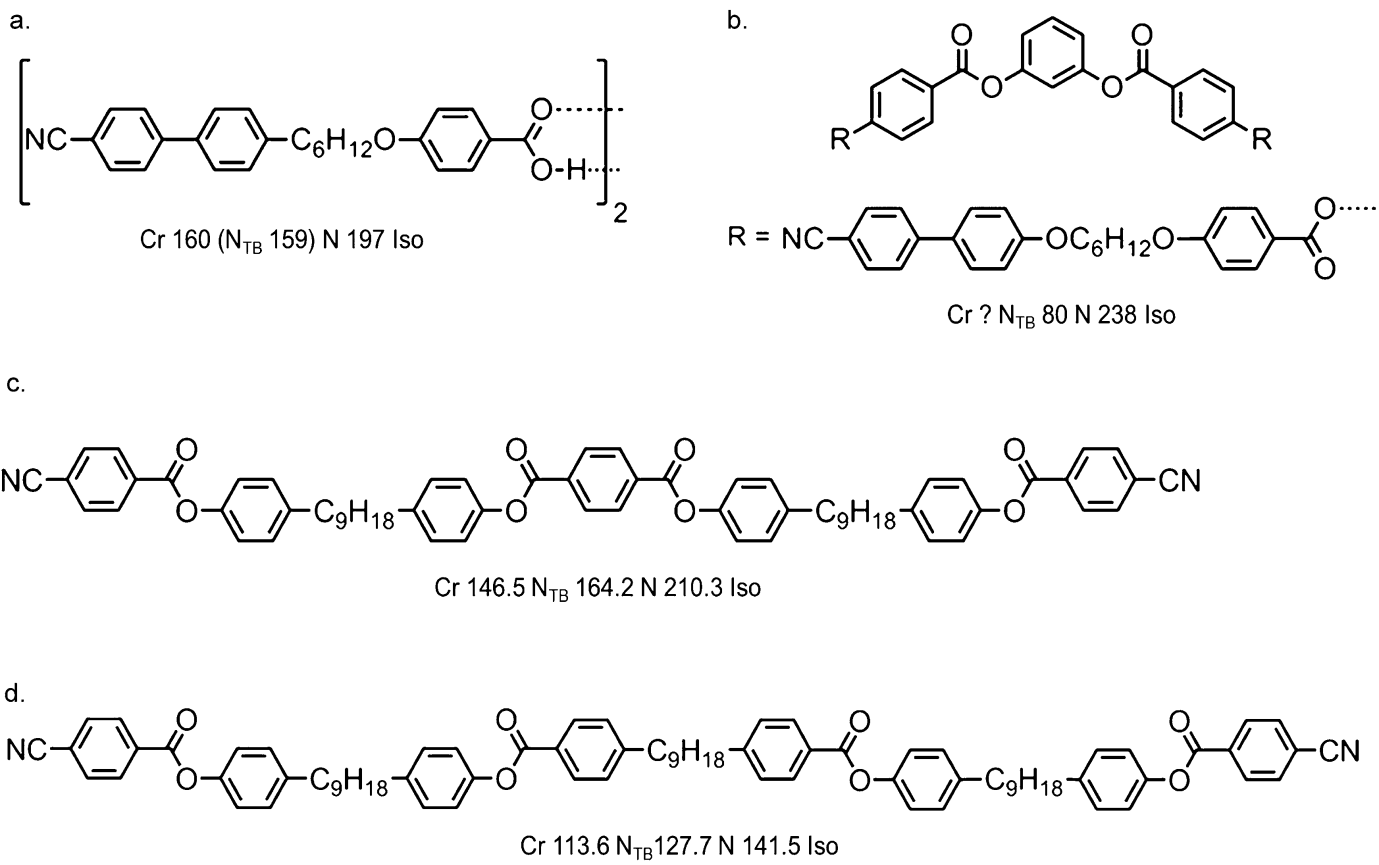
Oligomeric twist–bend nematogens known in the literature as of late 2016: a) the hydrogen bonded trimer CB6OCB;^32^ b) Wang's timer, note that a melting point was not reported;^47^ c) the trimer **T3_9_**;^49^ d) the tetramer **T4_9_**.^48^

The twist–bend nematic phase as exhibited by liquid‐crystalline dimers is seemingly fairly well understood in terms of the molecular factors that govern the incidence of this phase, and so our attention now turns to oligomeric materials that exhibit this mesophase. Jansze et al. reported in 2014 on a novel hydrogen‐bonded liquid‐crystalline trimer that also exhibited the twist–bend nematic phase (Figure 5 a).^32^ This material, known as 4‐[6‐(4′‐cyanobiphenyl‐4‐yl)hexyloxy]benzoic acid (CB6OBA), has an odd‐parity spacer unit—the homologous even‐parity material (CB5OBA) does not exhibit the N_TB_ phase, mirroring trends seen for dimers and bimesogens. Shortly after this Wang et al. reported a hybrid calamitic/bent–core trimer that exhibited the N_TB_ phase.^47^ This material, shown in Figure 5 b, with two cyanobiphenyl mesogenic units appended to a central resorcinol derived bent–core type mesogenic unit. In 2016 a methylene‐linked tetramer (**T4_9_** or RM1697, Figure 5 d) was reported by us; this material exhibits enantiotropic nematic and twist–bend nematic mesophases,^48^ as does the related linear trimer **T3_9_** (RM1698, Figure 5 c).^49^ Both **T3_9_** and **T4_9_** can be prepared in the same manner; an intermediate with one complete mesogenic unit and one half‐complete mesogenic unit bearing a phenol is esterified with an appropriate dicarboxylic acid to yield a trimer/tetramer.

**Figure 6:**
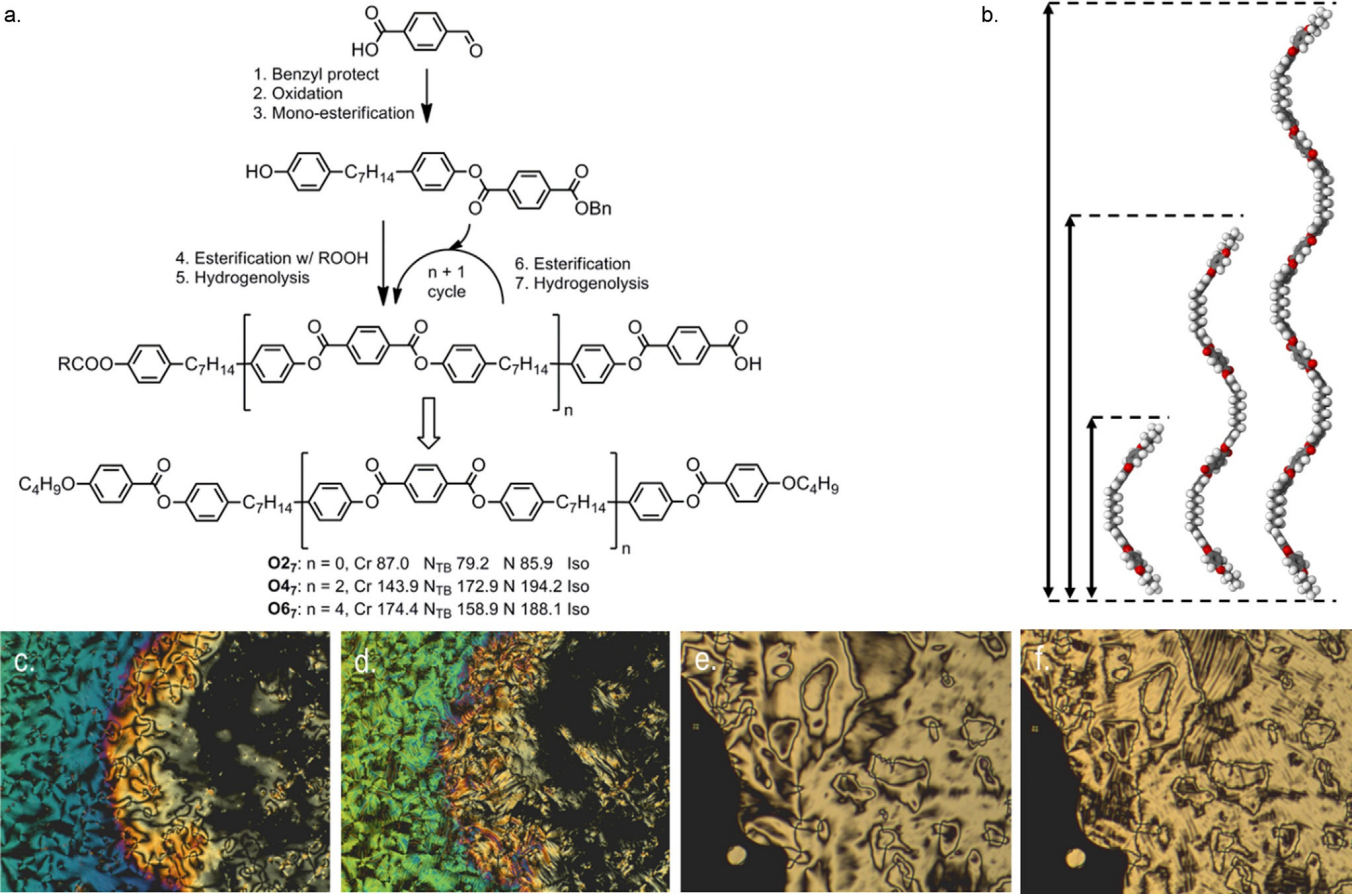
Oligomeric twist–bend nematogens: a) truncated synthetic route to **O4_7_** and **O6_7_**, with the transition temperatures of related dimer given alongside the two oligomers;^50^ b) comparison of the all‐*trans* geometries and end‐to‐end lengths obtained at B3LYP/6‐31G(d) for **O2_7_** (ca. 4 nm), **O4_7_** (ca. 8 nm) and **O6_7_** (ca. 12 nm); c) POM image of the nematic phase of **O4_7_** at 184 °C; d) the same region cooled into the N_TB_ phase of **O4_7_** at 169 °C; e) POM image of the *schlieren* texture of the nematic phase of **O6_7_** at 174 °C; f) approximately the same region of **O6_7_** cooled into the N_TB_ phase at 156 °C showing the blocky texture.^50^

It had been hypothesised that higher oligomers should exhibit the twist–bend nematic phase,^48^, ^49^ and whereas dimers, trimers and tetramers are readily accessible the synthesis of higher methylene‐linked oligomers has not been reported. We therefore devised a synthetic approach (Figure 6) to these materials that relies on an intermediate containing two half‐complete mesogenic units (4‐hydroxyphenyl and benzyl 4‐carboxyphenyl benzoate) separated by a spacer, in this case heptamethylene.^50^ Esterification of the free phenol with a suitable carboxylic acid followed by debenzylation by means of hydrogenolysis yields another carboxylic acid; this is then free to be esterified with another portion of the phenol/masked‐acid intermediate, extending the length of the molecule in a stepwise manner, providing essentially monodisperse oligomeric materials. To date we have used this approach to prepare a linear tetramer (**O4_7_**) and a linear hexamer (**O6_7_**), both of which exhibit the twist–bend nematic mesophase (Figure 6). Assuming threefold rotation—a simplification in itself—about each methylene unit the number of conformers of each oligomer is large enough to make study of the conformational landscape of these materials in their entirety (as opposed to truncated forms) challenging. While an abundance of liquid‐crystalline trimers and tetramers are known in the literature, higher oligomers are something of a rarity. It is believed that with refinement (and possibly automation) of the stepwise “*n*+1” approach we can prepare linear “oligomeric” materials of almost any length with a polydispersity approaching unity. However, as with polymers, there will exist some length scale at which globular shapes dominate over linear forms, and beyond this point the N_TB_ phase may not be observed.

**Figure 7:**
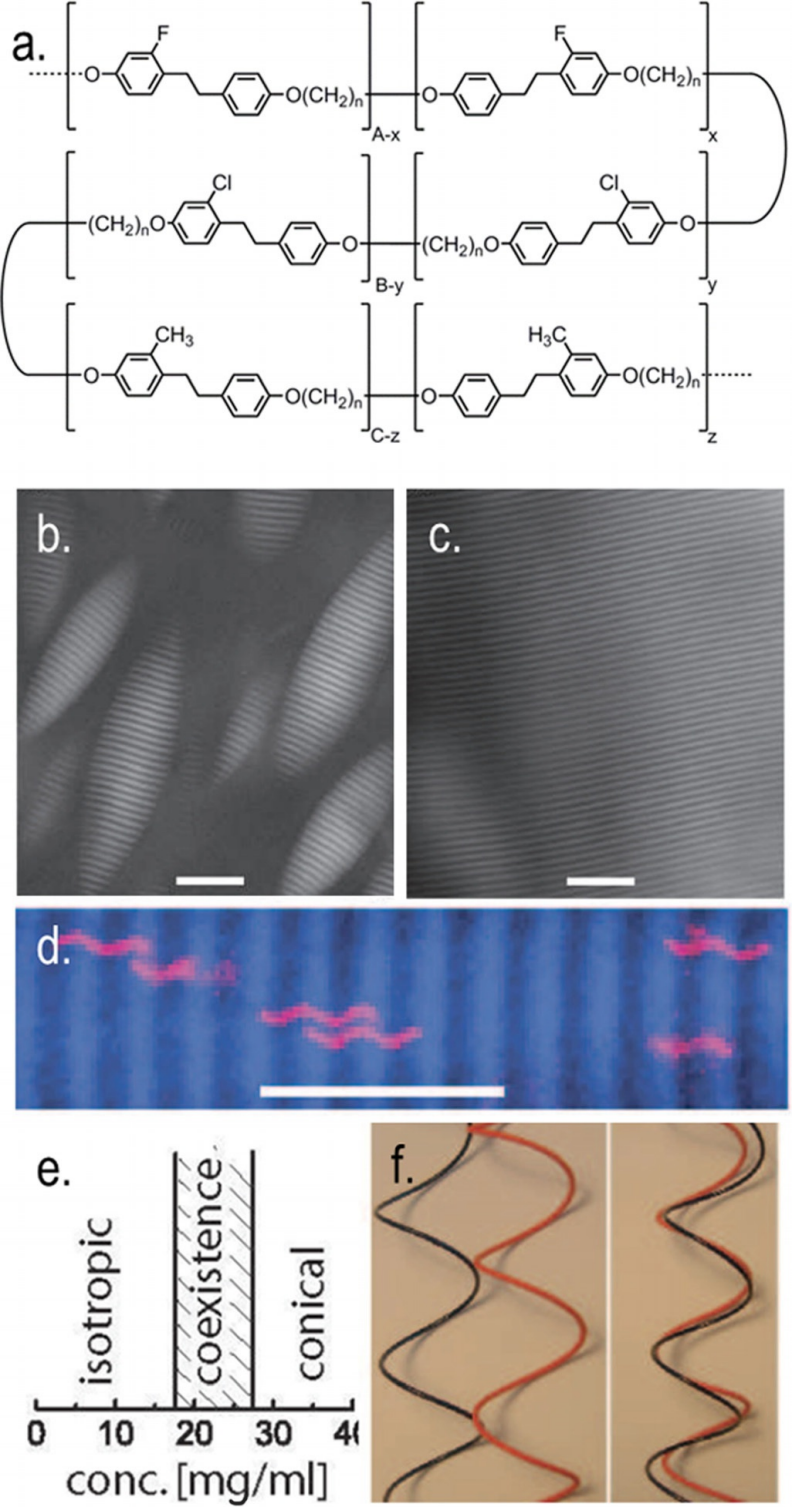
The N_TB_ phase on length scales beyond the molecule: a) molecular structures of copolymers (*n*=5, 7 or 9) reported by Ungar et al. that exhibit nematic to nematic transitions (denoted as N–N_2_ in the original paper). b), c) Polarised optical micrograph of the coexistence between the isotropic and conical nematic mesophase in flagella SJW1103 at 20 mg mL^−1^, scale bar is 20 μm; polarised optical micrograph of a macroscopically aligned sample of the conical nematic mesophase in flagella SJW1103 obtained by allowing a coexistent sample to phase separate. d) Fluorescently labelled flagella dissolved within the conical nematic phase of unlabelled flagella. e) The phase diagram of SJW1103 flagella expressed as a function of concentration (wt/vol). f) Illustration of the excluded volume between two helical rods out of phase (left) and in phase (right) with respect to one another. Reprinted Figure with permission from reference 54 copyright 2006 by the American Physical Society.

Liquid‐crystalline dimers and oligomers have long been considered as effective model compounds for semi‐flexible main‐chain liquid‐crystal polymers.^51^ It is therefore logical to postulate as to the existence of polymeric materials that exhibit the twist–bend nematic, as well as other modulated nematic phases exhibited by low‐molecular‐weight dimers. Long before the twist–bend nematic phase was topical, a series of methylene‐linked main‐chain polymers (Figure 7 a) were found to exhibit a nematic‐to‐nematic phase transition.^52^ After some speculation,^1^ subsequent reinvestigation of these materials indicates that the lower temperature nematic phase, denoted as N_2_ in the original paper, is in fact the twist–bend nematic phase.^53^

Barry et al. reported a first‐order phase transition driven by entropy in suspensions of helical flagella from an isotropic liquid into a liquid‐crystalline state with novel chiral symmetry (Figure 7).^54^ It was also demonstrated that achiral rods (in this case non‐helical flagella) do not show this phase, but rather exhibit a simple nematic. The optical textures are shown in Figure 7 and consist of a striped pattern with alternating light and dark regions, these correspond to differing director orientations. As shown in Figure 7 c, fluorescent labelling of flagella reveals that they are always in phase with one another and despite the lack of positional order there is long‐range “phase” ordering.^54^ It was proposed that the formation of this conical phase—as opposed to a simple nematic or chiral nematicphase—is driven by simple packing (steric) constraints; the excluded volume between two helices being significantly larger when they are out‐of‐phase with respect to one another (Figure 7 e, left) than when they are in phase (Figure 7 e, right) and so minimisation of the excluded volume dictates a preference for in‐phase packing leading to the emergence of the conical mesophase. Parallels exist between this lyotropic phase and the N_TB_ phase not only in terms of their shape‐driven origins, but also their properties. Helical flagella were observed to diffuse along the helical axis in a manner akin to a “nut on a bolt” with diffusion being significantly faster parallel to this axis with respect to perpendicular diffusion. Recent ^2^H NMR diffusiometry experiments have demonstrated similar anisotropic diffusion in the twist–bend nematic phase of CB7CB.^55^

## Summary and Outlook

In an achiral material the spontaneous breaking of mirror symmetry leads to domains of the N_TB_ phase of opposite handedness. By adding a small percentage of a chiral agent (< wt %) macroscopic (>200 μm^2^) domains of single handedness can be obtained, and these could be exploited through templating to give three‐dimensional nanostructured materials.^56^, ^57^ When used as a reaction solvent the twist–bend nematic phase may also present a more efficient method of chirality transfer than that afforded by conventional chiral liquid‐crystalline phases due to the short helical pitch, manifesting as a long chiral correlation length. Relative to dimers, there are relatively few examples of oligomeric materials known to exhibit the twist–bend nematic phase and this appears to be a logical direction for future research. Lastly we speculate, as others have done,^1^ that the entropy‐driven first‐order “conical nematic” phase exhibited by flagella may be a lyotropic analogue of the twist–bend nematic phase.

The molecular factors underpinning the twist–bend nematic phase in liquid‐crystalline dimers and bimesogens are now largely understood. The discovery of a linear relationship between *T*_NTB–N_ and *T*_N–Iso_ taken in conjunction with experimental demonstration of the importance of bend angle demonstrates that this mesophase is driven by gross shape, the minimisation of free or excluded volume and entropy. It may be possible to exploit the N_TB_ phase in display devices—provided that the difficulty in obtaining suitable alignment is overcome—and there are reports of fast electrooptic response for some materials, which occur near to the N→N_TB_ phase transition.^29^ Twist–bend nematic materials have been demonstrated to exhibit switchable reflection of light and this may find some applications.^58^, ^59^ Manipulation of the striped optical texture of the planar aligned N_TB_ phase has been demonstrated to be possible using applied AC fields, and this may find use in spatial light modulation.^60^

## Conflict of interest

The author declares no conflict of interest.
